# The effect of land-use on the diversity and mass-abundance relationships of understory avian insectivores in Sri Lanka and southern India

**DOI:** 10.1038/srep11569

**Published:** 2015-06-25

**Authors:** Rachakonda Sreekar, Umesh Srinivasan, Christos Mammides, Jin Chen, Uromi Manage Goodale, Sarath Wimalabandara Kotagama, Swati Sidhu, Eben Goodale

**Affiliations:** 1 Key Laboratory of Tropical Forest Ecology, Xishuangbanna Tropical Botanical Garden, Chinese Academy of Sciences, Menglun, Mengla, Yunnan 666303 CHINA; 2 National Centre for Biological Sciences, GKVK Campus, Bellary Road, Bangalore, Karnataka 560065 INDIA; 3 Field Ornithology Group of Sri Lanka, Department of Zoology, University of Colombo, Colombo 3, SRI LANKA; 4 Nature Conservation Foundation, 3076/5, 4th Cross, Gokulam Park, Mysore, Karnataka 570 002 INDIA; 5 University of the Chinese Academy of Sciences, Beijing, CHINA

## Abstract

Understory avian insectivores are especially sensitive to deforestation, although regional differences in how these species respond to human disturbance may be linked to varying land-use histories. South Asia experienced widespread conversion of forest to agriculture in the nineteenth century, providing a comparison to tropical areas deforested more recently. In Sri Lanka and the Western Ghats of India, we compared understory insectivores to other guilds, and to insectivores with different vertical strata preferences, both inside mixed-species flocks and for the whole bird community. Overall species richness did not change across the land-use gradient, although there was substantial turnover in species composition between land-use types. We found that the proportion of species represented by insectivores was ~1.14 times higher in forest compared to agriculture, and the proportion of insectivores represented by understory species was ~1.32 times higher in forests. Mass-abundance relationships were very different when analyzed on mixed-species flocks compared to the total community, perhaps indicating reduced competition in these mutualisms. We show that South Asia fits the worldwide pattern of understory insectivores declining with increased land-use intensity, and conclude that these species can be used globally as indicator and/or umbrella species for conservation across different disturbance time scales.

Anthropogenic habitat loss, degradation and fragmentation are perhaps the greatest current threats to biodiversity in the tropics today^1^. In the context of the ongoing biodiversity crisis, identifying species that are particularly vulnerable to anthropogenic disturbance, and traits that predispose them to vulnerability, is crucial to strategically plan conservation initiatives^2^. In birds, those species that are insectivorous and use the understory layer of stratified forests (hereafter referred to as ‘understory insectivores’) are especially vulnerable to anthropogenic change^3^,^4^,^5^.

A recent review^4^ found the mechanisms that make understory insectivores sensitive to human disturbance are complex and include the connection of these birds to unique features of the abiotic (microhabitat) or biotic (vegetation) rainforest understory environment, their low dispersal ability, and poor conditions for them in fragments (low prey abundance or high amount of nest predation). In contrast, frugivores and nectarivores may naturally range farther and take advantage of early successional habitat^6^,^7^, and granivores generally thrive in open landscapes^5^.These properties thus may make understory insectivores ‘indicator’ species for gauging the level of human disturbance in an area, and if they can be conserved, potentially ‘umbrella’ species whose protection can simultaneously preserve other kinds of biodiversity^8^.

Given that understory insectivores could have important roles to play in future conservation actions, it is important to fully understand the ways in which they respond to human disturbance, with more information required from under-represented areas in the world, and a better understanding of how they respond to different land-use histories. Sampling effort on understory insectivores is currently unequal throughout the world, with detailed data from some specific projects such as the Biological Dynamics of Forest Fragments Project in Brazil^9^,^10^, and more generally, a diversity of data from the Neotropics^4^, but with little data from other regions such as Asia and more specifically the Indian subcontinent (hereafter referred to as ‘South Asia’; but see ref^11^). Such sampling inequality could be troublesome if there are large regional differences in how understory insectivores respond to human disturbance. For example, in a recent meta-analysis, Bregman *et al.*^3^ found that insectivores were more vulnerable to fragmentation than other guilds in the tropics but not in temperate regions. The authors ascribe this result to life history differences between tropical and temperate birds, including low dispersal abilities and low densities of tropical birds^12^,^13^, but acknowledge that they did not explicitly incorporate differences in land-use history between regions into their analysis^3^. Regions can differ in the scale and intensity of forest conversion – i.e., in how degraded forests are and what matrixes are found between them – and also in the timing of the human disturbance^14^. As to timing, studies in recently fragmented areas will show different patterns compared to sites with a longer history of fragmentation, as recently fragmented areas will still retain some species that are bound for extinction, but will only go extinct over an extended time^15^,^16^.

South Asia is a particularly interesting area to investigate the relative persistence of understory insectivores compared to other guilds because large areas of forest were converted to agriculture in the nineteenth century^17^,^18^. Although the land-use history is not uniform (see study site description below), the fragmentation process is more than a century and a half old, quite different from the rapid, recent changes currently seen in other parts of the tropical world^19^. In areas with such long fragmentation histories, forest interior species may have already been driven to regional extirpation. In addition, because of the ecological isolation of the rainforests in the southern part of this region (the Western Ghats of India and Sri Lanka), it is thought that dispersal-limited species may have never reached there^20^, with low numbers particularly of babblers (Family Timaliidae), a family characteristic of tropical East Asian forests, many of which are understory specialists^21^. Because of this evolutionary and ecological history, it is possible that Sri Lanka and the Western Ghats are depauperate in understory insectivores, which could make it difficult to see current differences between guilds in their sensitivities to human disturbance. We test this hypothesis here, using data from a recent large scale study in the region, in which transects were located in three different land-use intensities: forests, buffer habitats consisting of degraded forest and agroforests, and areas of intensive agriculture^22^.

Beyond comparisons to other guilds, we also look at species interactions and the community structure of insectivores, as such community properties might be even more sensitive to human disturbance than the species themselves are^23^,^24^. Mixed-species flocks are a dominant form of the social organization of insectivorous birds in the region^25^, and it is possible that the flock system itself may respond to land-use changes, having consequences for the participating species^26^. Hence, we analyze the presence of insectivores inside flocks, as well as within the overall bird community. It has also been recently shown that the negative relationship between body size and population size within a community of insectivores (hereafter referred to as “mass-abundance scaling”) is impacted by habitat degradation^27^. If human disturbance leads to reduced resource (invertebrate) availability, mass-abundance scaling could be a particularly sensitive tool for detecting disturbance. For our study, we hypothesized that larger birds would be more abundant within forest and therefore the mass-abundance relationship would be more steeply negative in areas with high land-use intensity. We also compared the properties of mass-abundance scaling in the total bird community to those in mixed-species flocks, as such flocks have been described as mutualistic systems with reduced competition^28^,^29^. We hence hypothesized that there would be a weaker negative relationship between body size and abundance in mixed-species flocks than in the total bird community.

## Results

**Figure 1:**
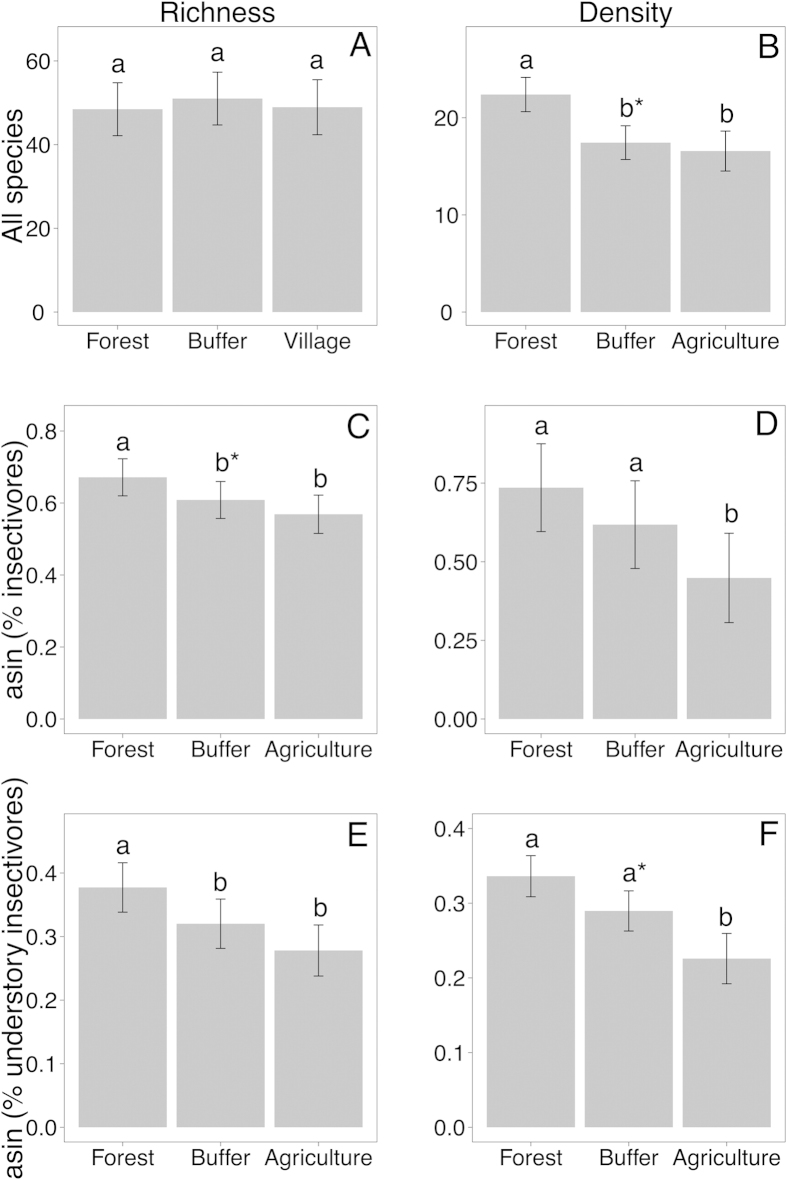
Changes in species richness and densities of all bird species (**A** & **B**), proportion of all species that were insectivores (**C** & **D**), and proportion of insectivores that inhabited the understory (**E** & **F**), across a gradient of land-use intensity in South Asia. Bars represent the expectations of the model fitted to the data and error bars represent standard errors. Bars with different letters are significantly different at the alpha level of P = 0.05, Bonferroni corrected; asterisks denote significance of 0.05 < *P* < 0.1.

**Figure 2:**
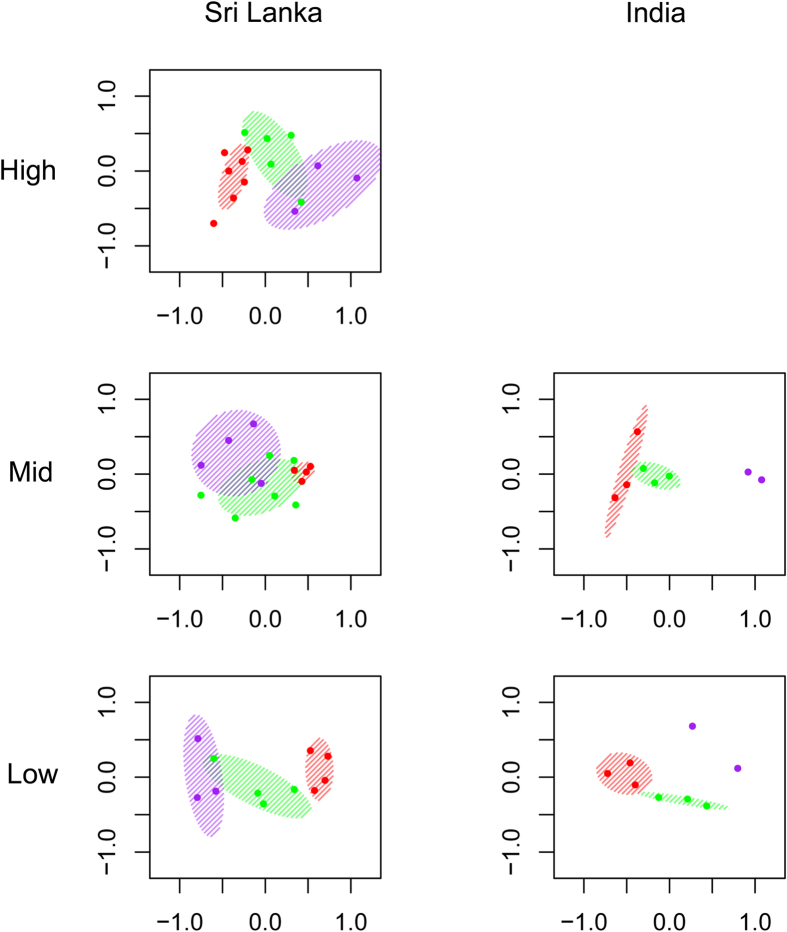
Nonmetric multidimensional scaling on the composition of bird communities in the different elevations of the two countries, using density data. Color codes: red for forest transects, green for buffer, purple for agriculture. Ellipses represent 99% confidence intervals calculated using standard errors by the *metaMDS* function of the *vegan* library of R; these are not calculable with less than three transects of one land-use type.

Avian species richness was surprisingly similar among land-use types (Table 1, Fig. 1A), while density was ~1.3 times higher in forests, in comparison to both buffer and agriculture (Fig. 1B). In considering this relative similarity in species richness, it should be remembered that there is considerable turnover in the species composition of these different land-use types (Sri Lanka: Deviance_(2,38)_ = 636.8, *P* *=* 0.01; India: Deviance_(2,13)_ = 593.8, *P* *=* 0.001; Fig. 2). For example, 39 of 166 species (23%) observed in forest were not observed in agriculture, and 28 of 155 species (18%) observed in agriculture were not observed in forest. Ordinations (NMDS) of the matrix of species densities at each transect shows clear compositional differences between forest, buffer, and agriculture, at every country/elevation combination (Fig. 2). Elevation was also a significant factor for species richness (see Table S1, Fig. S2) and composition (Sri Lanka: Deviance_(2,36)_ = 1273.9, *P* *=* 0.01; India: Deviance_(1,12)_ = 561.6, *P* *=* 0.001). There was also a significant interaction effect between land-use and elevation for species composition (Sri Lanka: Deviance_(4,34)_ = 287.2, *P* *=* 0.01; India: Deviance_(2,10)_ = 170.3, *P* *=* 0.001).

Diet influenced the richness and density of birds across the land-use gradient. The proportion of species that were insectivorous was affected by land-use (Fig. 1C; Table 1): forest transects had a ~1.14 times higher proportion of insectivorous species compared to agricultural transects, a proportion marginally significantly higher than buffer transects. The proportions of individuals that were insectivorous (Fig. 1D) was also strongly affected by land-use, with forests having proportionally ~1.64 times more individuals of these species than agricultural transects, and buffers also having ~1.38 times higher proportions of individuals being insectivorous than agricultural transects.

Vertical strata also influenced the species richness of insectivores. The proportion of insectivorous species that were classified as understory species declined strongly across the land-use gradient, with forests having ~1.18 times more understory species than buffers and ~1.32 times more than agricultural transects (Fig. 1E; Table 1). The proportion of individual insectivores that were members of understory species also decreased along the land-use gradient, with forests having proportionally ~1.48 times more individuals than agricultural transects (Fig. 1F; Table 1). However, for mixed-species flocks the proportion of understory insectivores to all insectivores did not change, neither in species richness nor in density (see Table 1).

**Figure 3:**
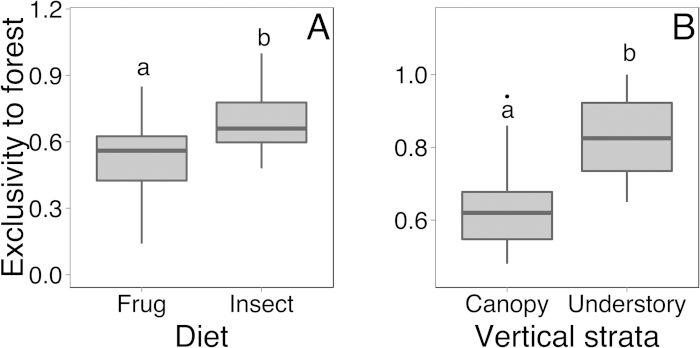
Forest interior species were more exclusive to forest (as measured by the percent of individuals estimated to be in the forest) if they were insectivorous, compared to frugivorous (**A**), or inhabited the understory, compared to the canopy (**B**).

When considering just forest interior species, insectivores were ~1.2 times more exclusive to forests than frugivores (Welch’s t_32.34_ = 2.58, *P* = 0.015, Fig. 3A). Understory insectivores tended to be ~1.3 times more exclusive to forests than other insectivores (Welch’s t_6.78_ = 2.32, *P* = 0.054, Fig. 3B). It should be noted that the sample size of understory insectivores for the last analysis is very small (six species, versus 14 canopy species); five of those six species are listed on the IUCN Red List (Table 2). Moreover, densities of seven out of nine Sri Lankan threatened endemic insectivore species were significantly reduced in agriculture compared to forest (Table 3).

**Figure 4:**
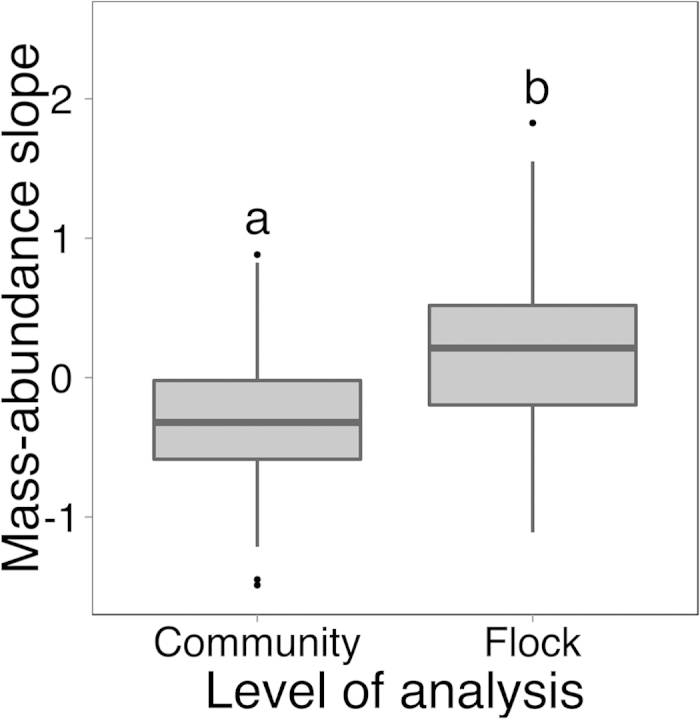
The slope of mass-abundance scaling for mixed-species flocks compared to the total bird community. N = 52 transects on which complete flocks were seen.

The slopes of mass-abundance scaling did not show consistent patterns with land-use (see Table 1). However, across all elevations, and within each land-use category, the slope of mass-abundance scaling was consistently higher in flocks, compared with the overall understory insectivorous community (except for agricultural habitats in the mid-elevations, which appeared to show no change). As expected, the slopes of mass-abundance scaling in the overall insectivorous community were largely negative (large species having lower relative abundances than small species) and the slopes for mixed-species flocks significantly higher (Fig. 4; paired t-test; *t*_*50*_ = −5.61, *P* < 0.01); indeed, the average slope for mass-abundance scaling within mixed species flocks was positive, indicating that larger species tended to occur more in flocks than outside of them.

## Discussion

We found that despite the overall species richness being quite similar across the land-use gradient in our South Asian region, densities were lower in agriculture compared to forest, and the proportion of insectivores, and particularly understory insectivores, dropped steeply from forest to buffer to agriculture (Fig. 1C–F). This result from all species is complemented by an analysis of only forest interior species, as defined by the literature, which showed that even within forest-preferring birds, diet and vertical strata influenced their exclusivity to forest (Fig. 3), with understory insectivores being particularly vulnerable. Importantly, birds outside of mixed-species bird flocks drove this result: flocks themselves remain relatively dense in buffer areas^22^, and the percentage of understory birds in them did not change across the land-use gradient. Unexpectedly, we found no detectable differences in the slopes of mass-abundance relationships in the different land-uses. Below we suggest that the large turnover in species across the anthropogenic land-use gradient made detecting community-wide patterns difficult. Overall, our results support the idea that avian insectivores are particularly vulnerable to land-use change throughout the world and in areas with different disturbance timescales^3^,^4^,^5^.

Our results support the finding from previous studies that species richness outside of forests in the Western Ghats / Sri Lankan biodiversity hotspot can be similar to species richness inside forests^20^,^30^,^31^,^32^. Our data suggests that high biodiversity extends even to some agricultural areas, although species composition shifts, with forest specialists having low abundance outside forests^11^,^30^,^33^. This pattern can be interpreted in different ways. One idea is that the number of forest specialists in the region is low because species with poor dispersal capabilities have not been able to reach the region, given the large distance to the closest rainforest area in Eastern India and Myanmar^20^. Another perspective is that the abundance of birds outside of forests is related to the long history of human alteration of forests in the region, with the idea that a mosaic of different land-uses, including traditional agroforestry techniques, can retain biodiversity in human-modified areas^31^. This later viewpoint also implies that birds may have had adequate time to evolve and adapt their habitat selection so as to persist in human-modified areas, a viewpoint not often advocated in conservation biology, but made plausible by recent discoveries of rapid evolutionary change in populations^34^. Further, given the long history of fragmentation, it is possible that forest specialists may have already been driven to extirpation (although we must admit we have no knowledge of documented extirpations in the region). The question then arises whether given these conditions insectivores will currently still be more sensitive to land-intensity than other guilds of birds.

Our results demonstrate that in this tropical area, which has a comparatively long history of fragmentation, avian understory insectivores continue to be more sensitive to human disturbance than other guilds. Our results might have been different had we included “grassland” species (species that almost exclusively use open habitat) as “understory insectivores”. However, while such species do occupy areas close to the ground, their preferred microhabitats – high sun areas – are very different from the low light environments usually preferred by understory species^35^. Also, we note that our forest interior analysis did not include such grassland species by definition. The result that understory insectivores are particularly vulnerable in the region is echoed by what forest bird species are Red Listed in the area^36^. For example in Table 2, it can be seen that 7 of 10 (70%) Sri Lankan endemic insectivores Red Listed are understory species; yet only 37 of 94 (39%) of the insectivores encountered in the study were classified as understory species (Table S1; a total of 9 understory insectivores [24%] were Red-listed). Our data is less plentiful for Western Ghats endemics; however, it is probable that similar trends exist there^37^. Hence for 150 years in the mountains of these areas these understory insectivores have persisted with small populations mostly confined to forests, and without modifying substantially their habitat selection. It is important to understand now the mechanisms behind this persistence, especially the movement patterns of the species to see what kinds of matrixes allow them to disperse and maintain gene flow^38^.

Contrary to our expectations, we were unable to detect consistent and significant differences in mass-abundance scaling across land-use intensification. The negative relationship between body size and population size is one of the most ubiquitous properties of ecological communities^39^, and is thought to be energetically determined^40^. Unlike Srinivasan^27^, who found more negatively steep mass-abundance scaling in understory insectivores in response to a relatively small gradient of habitat disturbance (from logging alone) in Northeast India, our gradient of land-use intensification is much larger and more drastic (see Fig. 2). Given that conversion from forest to agriculture results in a large shift in the bird community, it is conceivable that agricultural bird communities might be relatively intact, composed largely of what we defined as “grassland” species. In this situation, it is likely that mass-abundance scaling in agricultural landscapes reflects that of an intact open-country community, which is not expected to be different from mass-abundance scaling within forest communities. We urge future researchers to look for changes in mass-abundance scaling at a finer scale, especially within buffer habitats.

We also would like to highlight our strong result that mass-abundance scaling is less steep in mixed-species flocks (Fig. 4). Given that higher competition for resources can lead to steeper mass-abundance scaling from the loss or reduction of large species^27^, our results are consistent with the observation that flock participants enjoy increased access to resources through facilitative interactions and reduced competition^28^,^41^, and may even chose to join flocks of similarly sized birds^29^, a pattern previously noted in multiple flock systems from a single site^42^. The slope of mass-abundance scaling was particularly positive in those flocks lead by the relatively large Orange-billed Babbler (*Turdoides rufescens*) in Sri Lanka. Mixed-species flocks may be important in retaining bird species, particularly large ones, in human-modified areas, and further studies on how to retain them, perhaps targeting leader species, are required^22^.

In conclusion, we found that insectivores and particularly understory insectivores are particularly sensitive or vulnerable to land-use intensification in our study region of South Asia, which has a longer history of deforestation than other areas in the topics. More research is needed to determine the vegetation that allows understory insectivores to persist in this fragmented landscape^30^,^32^. Especially important are further studies on the movement ecology and conservation genetics of these birds, as studies have as yet been limited in this landscape to phylogeography^38^. However, the current state of knowledge makes it clear that understory insectivores are at an immediate risk of extinction^3^,^4^,^5^. Therefore, without delay, any unprotected forest fragments that have Red Listed understory avian insectivores, or the appropriate well-developed understory that these species prefer, should be a priority for conservation in South Asia and elsewhere.

## Methods

### Study sites

We sampled areas of wet evergreen forest in Sri Lanka and the Western Ghats of India. Large areas of the mountains and foothills in this region were converted to plantations of coffee and then tea toward the end of the 19^th^ century^17^,^18^; fragmentation has, however, been more recent in lowland Sri Lanka, as the market for lowland tea has expanded in the last half of the 20^th^ century^43^. Fifty-seven transects (forty 2-km, and the rest 1-km) were located over a 90 to 2180 m elevational gradient, in one of three land-use types: inside protected forests, on the boundary of reserves (“buffers”) in degraded forest and timber plantations, and in intensive agriculture. At each elevation we attempted to sample all three land-use types, so that they were spatially interspersed; see map in Fig. S1. In the study of Goodale *et al.*^22^, there are analyses of bird abundance and distribution at the level of specific land-use types (e.g., eucalyptus plantation, homegarden, etc.); here we concentrate on the more coarse level comparisons between forest, buffer and agricultural transects.

Forest habitats were mature evergreen rainforest. The majority of protected reserves included in this study were not systematically logged during the last half century (although roads may have been cut through them, n = 15 transects), while a minority were selectively logged during the 1970’s (n = 4 transects) and 1980’s (n = 2). By and large they are well protected now, although small scale extraction for firewood and polewood does occur.

Buffer habitats included degraded forest and timber plantations on the boundaries of protected reserves. Timber plantations, planted by the forest departments of the respective countries, and to a lesser extent private agencies, were primarily pine and eucalyptus, with teak in lowland India. In mid-elevation India buffer habitats also included shade-coffee and cardamom plantations retaining native canopy species^44^.

Transects in agriculture sampled primarily tea, but also included home gardens around human settlements, particularly in lowland Sri Lanka and India. Some fragments, usually of degraded forest, still occur in agricultural areas^45^.

Transects were placed on existing walking paths or roads. In most cases these were dirt paths, but 16 transects were on a sparsely traveled paved road at some point. Of the seven forest transects on paved roads, there were three for which the canopy was mostly continuous across the road. For the four others, and for one additional dirt logging road, the road may have formed an edge habitat or a barrier to dispersal^46^. However, the forests on either sides of the roads on these transects were continuous and alternative paths were unavailable.

### Bird data

Sri Lankan observations were collected by a team of two people; with one member of the team more experienced and the other less so and designated as the data recorder. Seven people in all worked on the project in Sri Lanka, working in different combinations, as long as one had good experience. In India, observations were mostly made by SS working by herself. All observers went through the same training protocol, organized by EG, which included exercises estimating distances. We used a variable radius transect method^47^, noting the presence of all birds, whether seen or heard, and their distance from the transect (the distance was estimated visually). We walked at a constant rate of approximately 1 km per hour, but stopped when we saw a mixed species flock (defined as more than one species of bird clearly moving together), which we watched for 5–15 min. We discarded any flock observations less than 5 min, and considered a flock observation to be ‘complete’ when we estimated we had recorded more than 80% of the individuals. Additional flock records were taken when walking back on the transect, if a flock had not been seen previously that day within 500 m.

Transect visits were staggered over the annual cycle, in both breeding and non-breeding seasons, with each transect visited an average of 7.2 times in one year. We collected a total of 34,847 observations of individual birds (note an erratum in Goodale *et al.*^22^: there were a total of 35,686 observations, but this includes mammals as well, which are not considered here) of 204 species, and 398 complete observations of flocks. The majority of the data was from Sri Lanka (27234 observations, 327 complete flocks).

### Species classification

To investigate the disturbance effects on avian species traits, we measured three life history and ecological traits. 1) Primary diet. Using data from Rasmussen and Anderton^48^ and BirdForum (http://www.birdforum.net), we categorized all species into one of five diet classes: insectivores, frugivores, carnivores, nectarivores and granivores (eating a majority of arthropods, fruit, vertebrates, nectar and seeds, respectively; Supplemental Appendix). 2) Vertical strata. Using the same sources, we categorized birds into one of the following four vertical strata: terrestrial, understory, midstory and canopy. These classifications were made using any mention in the sources of the relative vertical position of birds in a stratified forest; if the case of any inconsistencies among the sources, we used the position that was noted most during our fieldwork. Seventeen species that live almost exclusively in open landscapes, and hence have no data on vertical stratification, were designated as “grassland” species, and four species were designated as aerial specialists. During the analysis, we classified terrestrial and understory birds together as “understory” species and midstory and canopy species as “canopy” species (see Supplemental Appendix), due to the small sample sizes (in the two separate countries, a total of 14 ground species, 21 understory, 27 midstory, 28 canopy) and similar trends between the ground and understory species and between the midstory and canopy species. 3). Forest specialization. Using Ali and Ripley^49^ and Grimmett *et al.*^50^, we designated “forest interior” species as those species known to inhabit relatively undisturbed forest, and not to occur in open landscapes. Bird taxonomy follows Gill and Donsker^51^.

### Density estimation

Using DISTANCE, we adjusted our count data to take into account differing detectabilities of species. To better meet the assumptions of DISTANCE (i.e., 100% detection at 0 m), we took off 3 m from our estimated distances, removing the small strip of vegetation on either side of the path or road composed of unsuitable habitat for most birds, and used half normal models with cosine adjustments selected by Akaike Information Criterion (AIC) and 100 m truncation. If a species had more than 40 observations outside of flocks, we estimated its detectability, and if a species had more than 40 such observations in each of the three land-uses, we estimated its detectability stratified by land-use. Species with less than 40 observations in total were given the detectability of the average species. Flocks were assumed to be detected as a group, and flock detectability was stratified by land-use and depending on whether the flock included the easily detectable (noisy) and numerous leading species, the Orange-billed Babbler, *Turdoides rufescens*, or not. Estimated birds / flocks were then divided by the sampling effort (in ha) for each transect. For species richness we used rarefaction to estimate how many species would have been seen on each transect had the sampling been the same as the least visited transect.

### Community-level analysis

Our primary analysis investigated the distribution and density of all species across land-use types, and then what proportion of this community are insectivores and more specifically understory insectivores. To examine species richness and overall densities in different land-uses, we used general linear mixed models, using the ‘*lmer*’ function of the *lme4* package^52^ in the program R [R Core Team, version 3.1.2, 2014], with a global model that had country as a random effect, and land-use, elevation (coded as low [0-500 m asl], middle [800–1300 m asl] and high [1500–2180 m asl]) and a landuse-elevation interaction as fixed effects. We did not find spatial autocorrelation among model residuals using Moran’s I tests (*P* > 0.1), and therefore did not make any spatial corrections to the original models; to see differences between countries, see Fig. S3 and S4. We used a backwards elimination procedure to sequentially simplify the model if a fixed effect was not significant, with the importance of fixed effects determined using likelihood ratio tests^52^. To estimate the significance of model parameters, we resampled 1000 times from the posterior estimates^53^, using the ‘*sim*’ function of the *arm* package, and calculated a two-tailed *P*-value following the method described in Bagchi *et al.*^54^. The model parameters were considered significant at *P* < 0.05 and marginally significant at 0.05 < *P* < 0.1; significance levels were adjusted using Bonferroni correction for multiple comparisons.

To determine the differences in community composition between land-use types and elevation gradients, we used multivariate generalised linear models with binomial error structure using the ‘*manyglm’* function in *mvabund* package on species presence-absence data^55^. We specified binomial regression in our models, and included land-use type, elevation and their interaction as predictors. Significance was assessed using 999 permutations of Monte Carlo tests and we present the Deviance and *P* values. We built two separate models for India and Sri Lanka. We used non-metric dimensional scaling (NMDS) to visualize composition data.

To determine whether being insectivorous makes species more sensitive to land-use intensity, or whether being an understory species makes an insectivore more sensitive, we calculated the proportion of species (at the transect level) composed of these diet and vertical stratum guilds. Because some of our analysis were continuous measurements (i.e., DISTANCE-adjusted densities) we did not use generalised linear mixed models, which are not appropriate for such data (for example, the binomial distribution best fits a ratio of counts), but rather arcsine transformed the proportion and conducted a general linear mixed model with a Gaussian distribution^56^.

A further analysis at the community level focused on mixed-species flocks. Because it is known that mixed-species flocks are mostly insectivorous in this region^57^, we did not ask analyze what percent of them were insectivores. But we did ask what percent of those species in flocks were understory insectivores, and then investigated whether this percentage (arcsine transformed) changed based on land-use type, as above.

### Species level analysis

We also conducted a species-level analysis that concentrated on forest interior species and endemic species. Using data from all the forest interior species, we asked whether diet (insectivores/frugivores; we were unable to test for other diet guilds because of low sample sizes) influenced the exclusivity of a species to forest (defined as the proportion of the overall density of a species that was recorded in forests), or if vertical strata influenced the exclusivity of insectivores to forest. For this analysis, we only included species over a certain sample size (0.01 birds/ha on average on the 21 forest transects), so that low sample sizes would not affect the reliability of proportional data. We arcsine transformed forest exclusivity and then with a Welch’s t-test investigated whether insectivorous species were more exclusive to forests than species with other diets, and for insectivorous species, whether understory species where more exclusive to forests than canopy species.

When we found that the endemic forest interior species that were understory insectivores in our region are usually listed as threatened by the IUCN^36^, we went a step further and investigated whether the abundances of these species changed due to land-use type. In this analysis we focused on Sri Lankan endemics, because we have the most powerful data on them. We used Kruskal-Wallis tests to determine whether the densities of endemic and threatened Lankan insectivores differed across land-use types, and Bonferroni-adjusted Wilcoxon tests for multiple comparisons when necessary.

### Mass-abundance scaling analysis

We examined the impact of land-use type and elevation on mass-abundance scaling, both in the overall understory insectivorous bird community, as well as mixed-species flocks. Only insectivorous species were included in these analyses. Further, canopy and aerial species were excluded from the analysis because they are not likely to compete with understory species for resources. Body mass data for most species was drawn from Dunning^58^. We were unable to obtain information on the body masses of seven species from the literature. For three of these species we used our own unpublished data, and for the remaining species, we found a similarly sized congeneric (as indicated from head to tail measurement) and used the weight of that species to estimate the missing data (see Supplemental Appendix).

The upper bound of the triangular mass-abundance relationship was estimated using quantile regression in the package *quantreg*^59^. To compare the impact of land-use type on mass-abundance scaling in flocks and the overall insectivorous bird community, we analyzed mass-abundance slopes at the transect level for (a) the entire understory insectivorous bird community, and (b) an ‘average’ flock composition at each transect that was the arithmetic mean of the abundance of different species across all flocks encountered on the transect. Mixed models investigating the effect of both land-use, elevation and their interaction were fitted as above.

## Additional Information

**How to cite this article**: Sreekar, R. *et al.* The effect of land-use on the diversity and mass-abundance relationships of understory avian insectivores in Sri Lanka and southern India. *Sci. Rep.* **5**, 11569; doi: 10.1038/srep11569 (2015).

## Supplementary Material

Supplementary Information

## Figures and Tables

**Table 1:** Results of mixed-models with fixed factors of land-use, elevation and their interaction, and random factor of country.

**Response Variable**	**Specific Category**	***X***^***2***^_***2***_	**P**	**Multiple Comparisons**^1^	**Parameter Estimates**	**CI (2.5%)**	**CI (97.5%)**	**P**
All Species	Species Richness	0.58	0.74					
All Species	Density	7.78	0.02	F vs. B	−4.88	−9.60	−0.81	0.057
				F vs. A	−6.05	−10.64	−1.23	0.034
Proportion of Insectivores	Species Richness	12	0.002	F vs. B	−0.06	−0.11	−0.009	0.08
				F vs. A	−0.09	−0.12	−0.05	0.006
Proportion of Insectivores	Density	20.04	< 0.0001	F vs. A	−0.28	−0.41	−0.16	< 0.001
				B vs. A	−0.17	−0.29	−0.05	0.021
Proportion of Understory	Species Richness	17.77	< 0.0001	F vs. B	−0.06	−0.09	−0.01	0.021
				F vs. A	−0.1	−0.14	−0.06	< 0.001
Proportion of Understory	Density	6.41	0.04	F vs. A	−0.11	−0.16	−0.06	0.003
				B vs. A	−0.06	−0.11	−0.001	0.069
Flocks: Proportion of Understory	Species Richness	1.33	0.51					
Flocks: Proportion of Understory	Density	3.6	0.16					
Mass-Abundance Slopes	All Species	1.15	0.56					
Mass-Abundance Slopes	Flocks	0.87	0.64					

We present parameter estimates on the scale of the linear predictor with their 95% confidence intervals for the land-use term here; for the elevation and interaction terms, see Table S1. Models were sequentially reduced when fixed effects were non-significant. Multiple comparisons were Bonferroni corrected, and only significant multiple comparisons are shown.

^1^Abbreviations: F = Forest; B = Buffer; A = Agriculture.

**Table 2:** Endemic Sri Lankan insectivores on the IUCN Red List (2013), and their vertical strata. Species followed by an asterisk were included in the analysis of the exclusivity to forests of forest interior species.

**Scientific name**	**English name**	**Vertical Strata**	**Designation**
Myophonus blighi*	Sri Lanka Whistling Thrush	Ground	EN
Zoothera imbricata*	Sri Lanka (Scaly) Thrush	Ground	NT
Geokichla spiloptera*	Spot-winged Thrush	Ground	NT
Garrulax cinereifrons*	Ashy-headed Laughing	Understory	VU
*Centropus chlororhnychos*^1^	Green-billed Coucal	Understory	VU
*Eumyias sordida*^2^	Dull-blue Flycatcher	Understory	NT
Elaphrornis palliseri*	Sri Lanka Bush Warbler	Understory	NT
Urocissa ornata*	Sri Lanka Magpie	Midstory	VU
Turdoides rufescens*	Orange-billed Babbler	Midstory	NT
Phaenicophaeus pyrrhocephalus*	Red-faced Malkoha	Canopy	VU

^1^Species did not have adequate sample size.

^2^Species not considered an forest interior species.

**Table 3:** Responses of Sri Lankan endemic and threatened insectivore species to land-use type.

**Species**	***X***^***2***^	**P**	**Multiple Comparisons**	**W**	**P**	**Response**
*M. blighi*^1^	-	-	-	-	-	Positive
*Z. imbricata*^2^	-	-	F vs. B	70.5	0.012	Positive
*G. spiloptera*^3^	15.56	<0.001	F vs. B	37	0.003	Positive
			F vs. A	136	0.002	Positive
			B vs. A	85	1	NS
*G. cinereifrons*^2^	-	-	F vs. B	87	0.15	NS
*E. sordida*^3^	1.25	0.53	-	-	-	NS
*E. palliseri*^3^	2.02	0.36	-	-	-	NS
*U. ornata*^3^	10.62	0.005	F vs. A	58	0.04	Positive
			B vs. A	127	0.01	Positive
			F vs. B	94.5	1	NS
*T. rufescens*^3^	8.34	0.015	F vs. A	61	0.05	Positive
			B vs. A	119	0.04	Positive
			F vs. B	92	1	NS
*P. pyrrhocephalus*^2^	-	-	F vs. B	87	0.06	Positive

The *X*^*2*^ values are derived from Kruskal-Wallis test. Multiple comparisons were made using Wilcoxon test and Bonferroni corrected. Positive response indicates increase in densities in more forested habitats. Tests were simplified if species did not occur in one of the three habitats, and no tests were conducted if species occurred only in a single land-use type.

^1^Species that were recorded in forests only. No tests were conducted.

^2^Species that were recorded in forests and buffers only. Only Wilcoxon tests were conducted.

^3^Species that were recorded in all three land-use types. Kruskal-Wallis tests were conducted and multiple comparisons were made using Wilcoxon test, and Bonferroni corrected.
