# Supplementary material for: The effect of land-use on the diversity and mass-abundance relationships of understory avian insectivores in Sri Lanka and southern India
Source: Sci Rep. 2015 Jun 25;5:11569. doi: 10.1038/srep11569 (PMC4479823; doi:10.1038/srep11569)
Supplement: Supplementary Information [file srep11569-s1.doc]

**The effect of land-use on the diversity, abundance, and mass-abundance relationships of understorey avian insectivores in Sri Lanka and southern India.**

Rachakonda Sreekar, Umesh Srinivasan, Christos Mammides, Jin Chen, Uromi Manage Goodale, Sarath Wimalabandara Kotagama, Swati Sidhu and Eben Goodale

**Supplemental Information**

Table S1…………………………………………………………………………………….Pages S1-S2

Supplemental Figure Legends…………………………………………………..………….Page S3

Figure S1…………………………………………………………………..………………..Page S4

Figure S2……………………………………………………………………………………Page S5

Figure S3……………………………………………………………………………………Page S6

Figure S4……………………………………………………………………………………PageS7

Appendix……………………………………………………………………………………Pages S8-S16

Table S1. Results of mixed-models: elevation and landscape effects only (for land-use effect, see Table 1). We present parameter estimates on the scale of the linear predictor with their 95% confidence intervals. Models were sequentially reduced when fixed effects were non-significant. Multiple comparisons were Bonferroni corrected, and only significant multiple comparisons are shown.

|  |  |  |  |  |  |  |  |  |  |
| --- | --- | --- | --- | --- | --- | --- | --- | --- | --- |
| Response Variable | Specific | Factor | *X^2^***^1^** | P | Multiple | Parameter | CI | CI | P |
|  | Category |  |  |  | Comparisons^2^ | Estimates | (2.5%) | (97.5%) |  |
|  |  |  |  |  |  |  |  |  |  |
| All Species | Species Richness | Elevation | 31.26 | < 0.0001 | L vs. M | -10.43 | -16.04 | -5.12 | < 0.001 |
|  |  |  |  |  | L vs. H | -20.06 | -26.47 | -13.42 | < 0.001 |
|  |  |  |  |  | M vs. H | -9.49 | -15.98 | -2.61 | 0.015 |
|  |  | Interaction | 0.96 | 0.91 |  |  |  |  |  |
| All species | Density | Elevation | 5.15 | 0.06 | L vs. H | 5.59 | 2.24 | 8.69 | 0.014 |
|  |  |  |  |  | M vs. H | 3.72 | 0.64 | 6.52 | 0.055 |
|  |  | Interaction | 2.28 | 0.68 |  |  |  |  |  |
| Proportion of Insectivores | Species Richness | Elevation | 47.51 | < 0.0001 | L vs. M | 0.08 | 0.04 | 0.12 | 0.001 |
|  |  |  |  |  | L vs. H | 0.17 | 0.13 | 0.22 | < 0.001 |
|  |  |  |  |  | M vs. H | 0.09 | 0.04 | 0.13 | 0.002 |
|  |  | Interaction | 3.78 | 0.43 |  |  |  |  |  |
| Proportion of Insectivores | Density | Elevation | 16.32 | 0.0007 | L vs. H | 0.25 | 0.11 | 0.38 | 0.006 |
|  |  |  |  |  | M vs. H | 0.21 | 0.08 | 0.33 | 0.012 |
|  |  | Interaction | 3.47 | 0.48 |  |  |  |  |  |
| Proportion of Understorey | Species Richness | Elevation | 15.45 | 0.0004 | L vs. M | 0.07 | 0.03 | 0.11 | 0.003 |
|  |  |  |  |  | L vs. H | 0.08 | 0.04 | 0.13 | 0.003 |
|  |  | Interaction | 2.72 | 0.60 |  |  |  |  |  |
| Proportion of Understorey | Density | Elevation | 10.68 | 0.004 | M vs. H | -0.34 | -0.41 | -0.26 | 0.003 |
|  |  | Interaction | 4.66 | 0.32 |  |  |  |  |  |
| Flocks: Proportion of  UUUnderstorey | Species Richness | Elevation | 3.91 | 0.14 |  |  |  |  |  |
| Understorey |  | Interaction | 6.89 | 0.14 |  |  |  |  |  |
|  |  |  |  |  |  |  |  |  |  |
|  |  |  |  |  |  |  |  |  |  |
| Response Variable | Specific | Factor | *X^2^***^1^** | P | Multiple | Parameter | CI  (2.5%) | CI  (97.5%) | P |
|  | Category |  |  |  | Comparisons^2^ | Estimates | (2.5%) | (97.5%) |  |
|  |  |  |  |  |  |  |  |  |  |
| Flocks: Proportion of | Density | Elevation | 9.01 | 0.01 | L vs. H | -0.11 | -0.19 | -0.02 | 0.039 |
| Understorey |  | Interaction | 5.99 | 0.19 |  |  |  |  |  |
| Mass-Abundance Slopes | Total Bird Community | Elevation | 21.18 | < 0.001 | L vs. H | 0.77 | 0.46 | 1.06 | 0.006 |
|  |  |  |  |  | M vs. H | 0.47 | 0.20 | 0.75 | 0.012 |
|  |  | Interaction | 4.96 | 0.29 |  |  |  |  |  |
| Mass-Abundance Slopes | Flocks | Elevation | 18.47 | < 0.001 | L vs. H | 0.67 | 0.35 | 1.01 | 0.006 |
|  |  |  |  |  | M vs. H | 0.56 | 0.28 | 0.81 | 0.003 |
|  |  | Interaction | 18.56 | 0.0009 |  |  |  |  |  |
|  |  |  |  |  |  |  |  |  |  |

^1^ The degrees of freedom are 2 for the elevation term and 4 for the interaction term.

^2^ Abbreviations: L = Low (0 – 500 m asl); M = Middle (800 – 1300 m asl); H = High (1500-2180 m asl).

Supplemental Figures

Figure S1. Transects (n=57) from which observations were made in Sri Lanka and India over an altitudinal gradient (as published in Goodale et al. 2014). Transects through forests are blue, those in “buffer” lands (degraded forests or plantations) at the border of forests are red, and those in agricultural lands are yellow. Beige colored areas are between 1000-1500 m asl, with dark brown above that and green and blue below. The width of the transect is not to scale. Topographical data are void-filled seamless SRTM data V1, 2004, from International Centre for Tropical Agriculture (CIAT), available from the CGIAR-CSI SRTM 90m database at [http://srtm.csi.cgiar.org](http://srtm.csi.cgiar.org/). A: Western sector of the Sinharaja World Heritage Reserve; B: eastern sector of Sinharaja; C: Nuwara Eliya region; D: Thattekad Reserve; E: Anamalai Hills.

Figure S2. Changes in species richness (A) and densities (B) of all species across an elevation gradient in South Asia. Points represent the expectations of the model fitted to the data and error bars represent the standard errors.

Figure S3. Data for Figure 1, but presenting information only for Sri Lanka.

Figure S4. Data for Figure 1, but presenting information only for India.

Figure S1


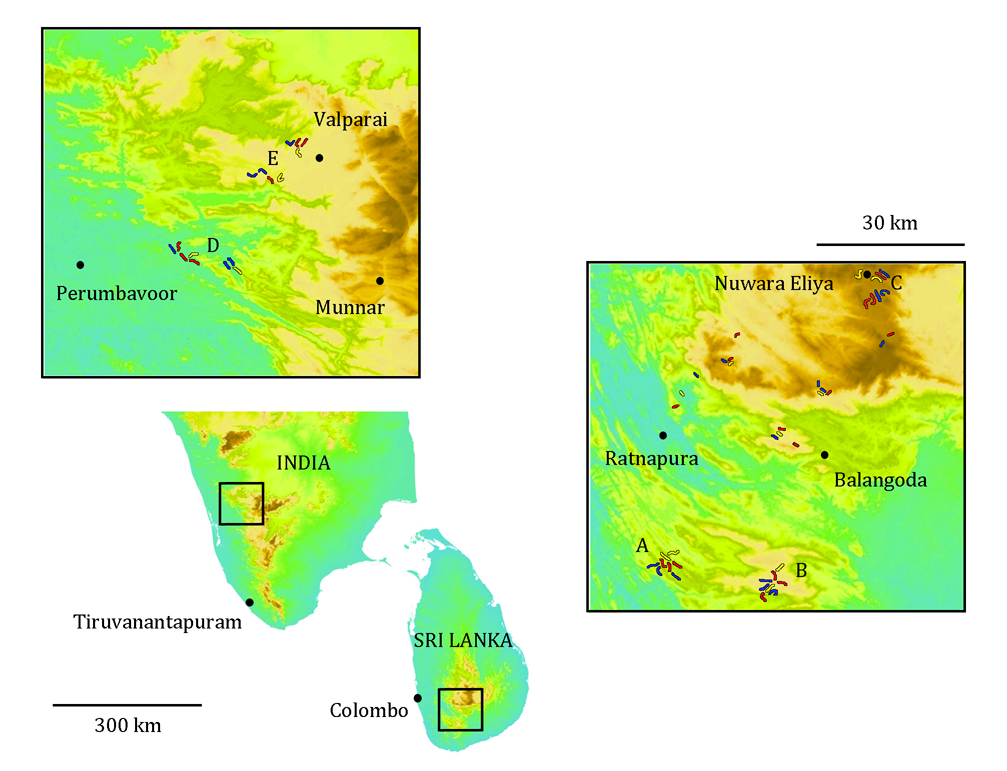


Figure S2


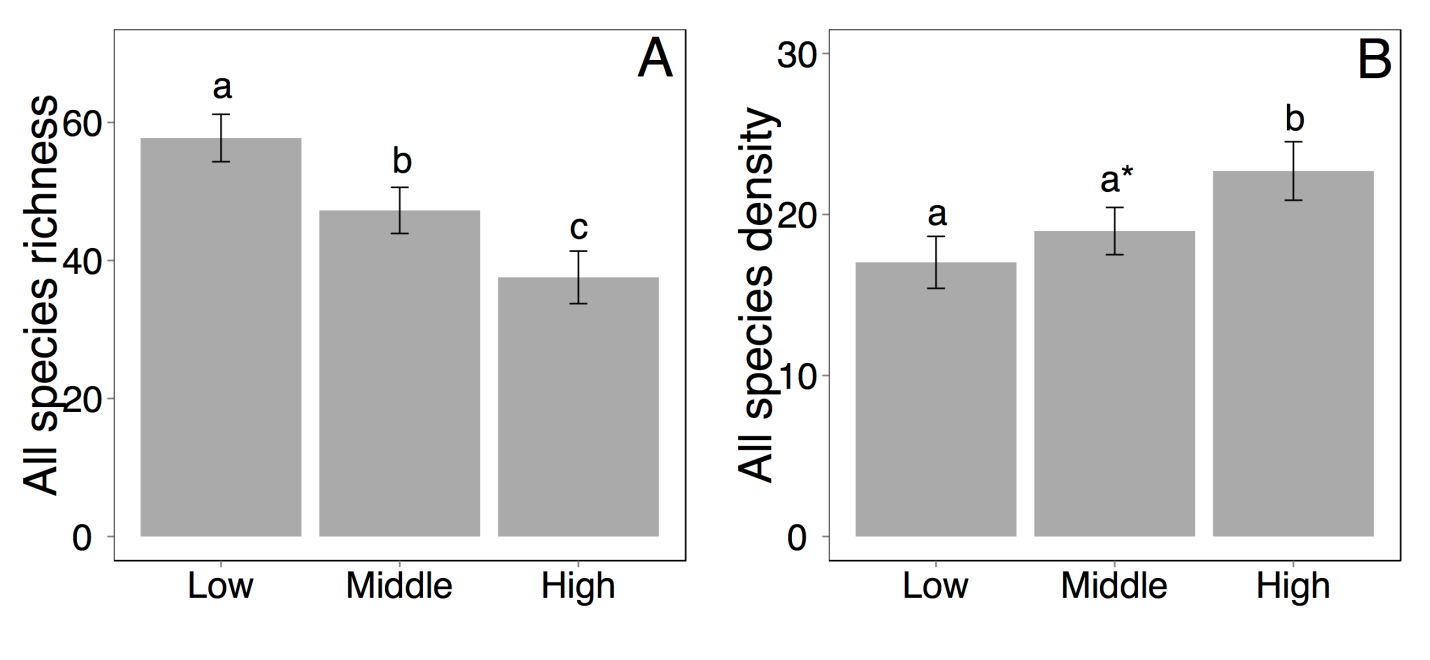


Figure S3


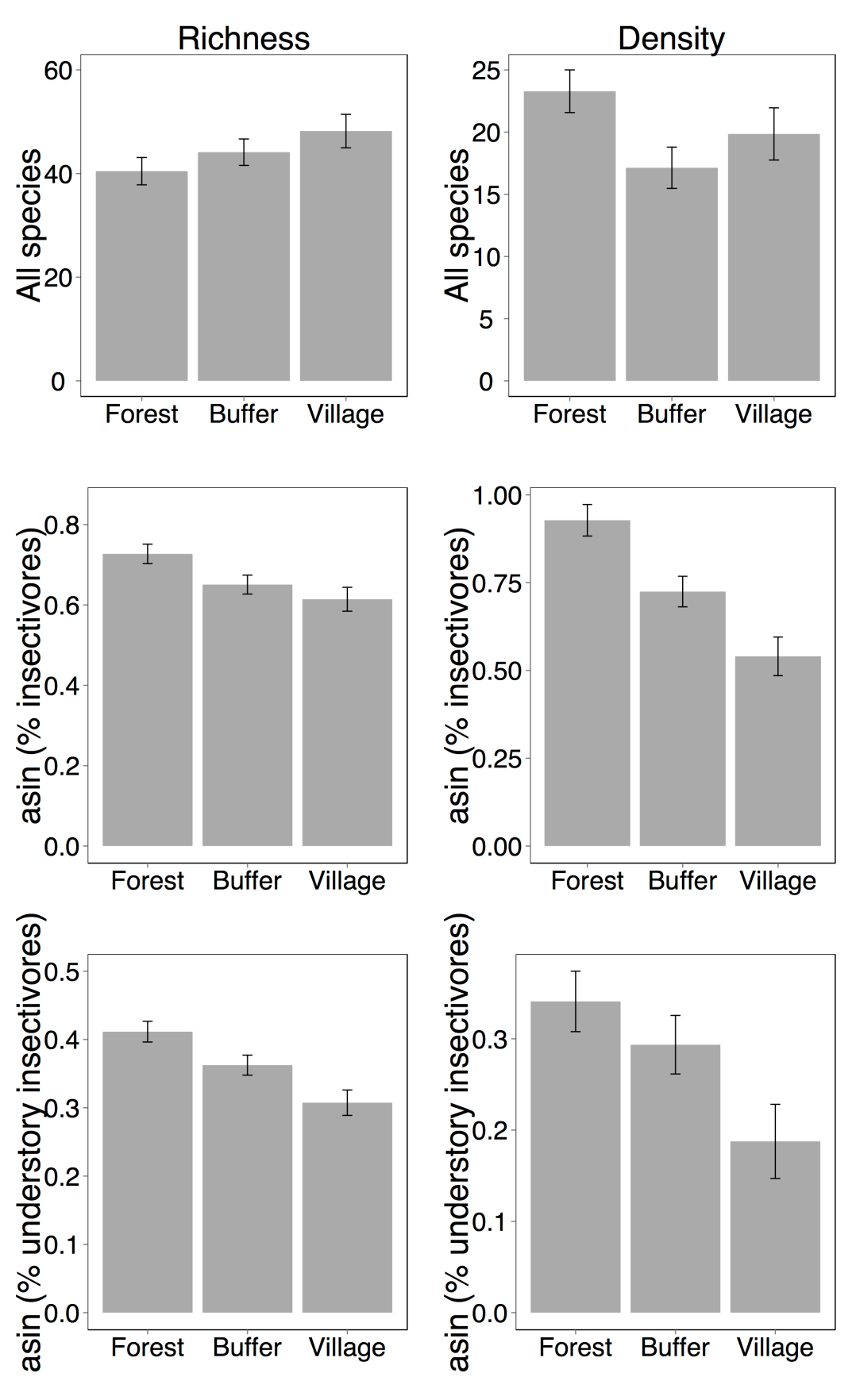


Figure S4


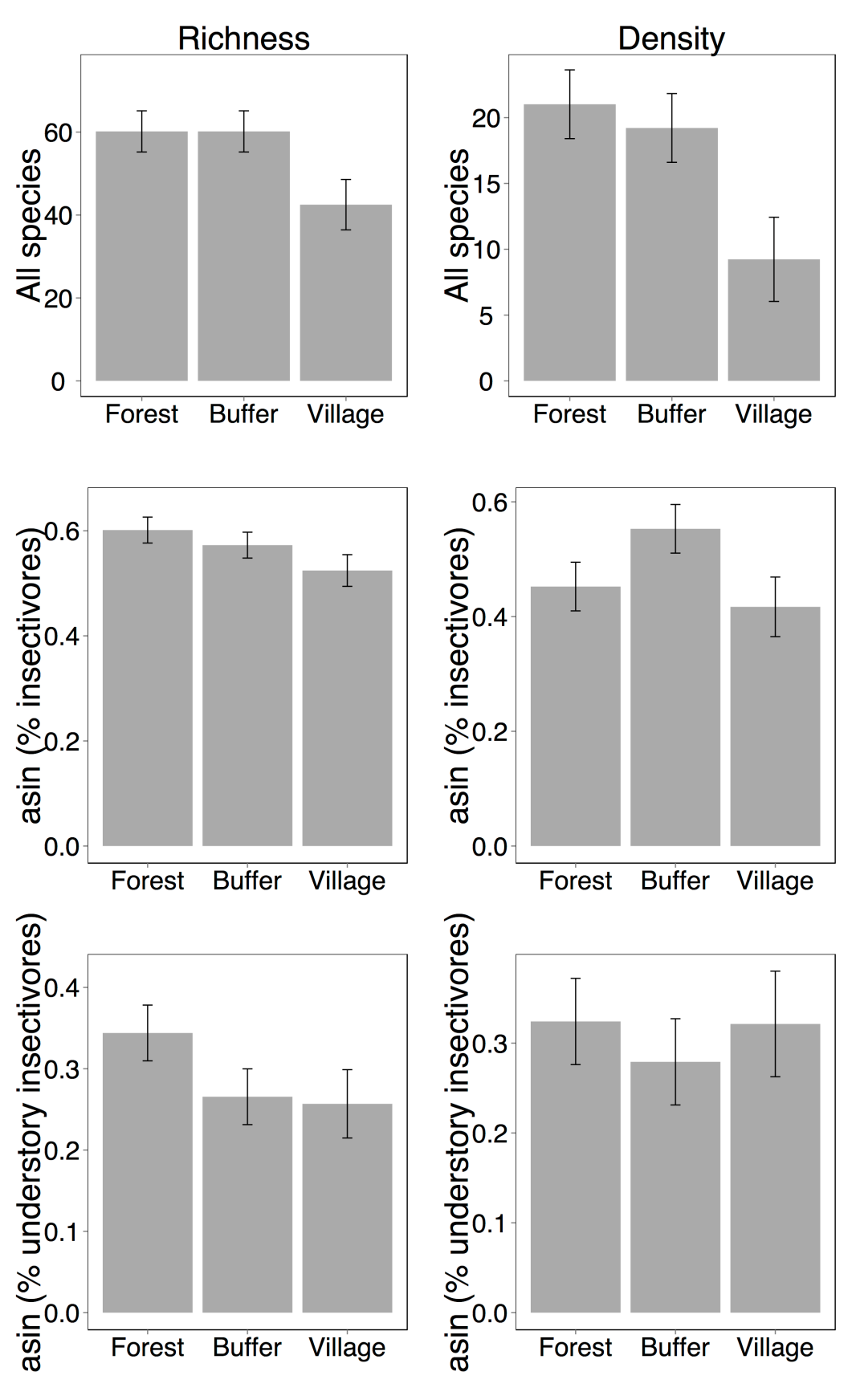


**Appendix.** All bird species recorded in this study in Sri Lanka and the Western Ghats of India: their diet, vertical strata, and mass. We provide masses only for insectivorous species that were not aerial or canopy species, as these were the only species included in the mass-abundance analysis. For species where mass was not available from Dunning (2008), we used our own unpublished data (three species) or estimated mass from a similarly sized congeneric (see “Source”). English names of species listed on the IUCN Red-list are bolded, and followed by an asterisk if they were classified as forest interior species. Taxonomy follows [Gill and Donsker (2013)](#_ENREF_15).

| **Family** | **English name** | **Scientific Name** | **Diet^1^** | **Vert^2^** | **Mass** | **Source** |
| --- | --- | --- | --- | --- | --- | --- |
| Phasianidae |  |  |  |  |  |  |
|  | Red Spurfowl | *Galloperdix spadicea* | G | NA | NA |  |
|  | Sri Lanka Spurfowl* | *Galloperdix bicalcarata* | G | NA | NA |  |
|  | Grey Junglefowl | *Gallus sonneratii* | G | NA | NA |  |
|  | Sri Lanka Jungle Fowl* | *Gallus lafayetii* | G | NA | NA |  |
| Anatidae |  |  |  |  |  |  |
|  | Lesser Whistling Duck | *Dendrocygna javanica* | F | NA | NA |  |
| Ardeidae |  |  |  |  |  |  |
|  | Malayan Night Heron | *Gorsachius melanolophus* | C | NA | NA |  |
|  | Indian Pond Heron | *Ardeola grayii* | C | NA | NA |  |
|  | Cattle Egret | *Bulbucus ibis* | I | grass | 366 |  |
|  | Purple Heron | *Ardea purpurea* | C | NA | NA |  |
|  | Greater Egret | *Ardea alba* | C | NA | NA |  |
|  | Intermediate Egret | *Egretta intermedia* | C | NA | NA |  |
| Phalacrocoracidae |  |  |  |  |  |  |
|  | Little Cormorant | *Microcarbo niger* | C | NA | NA |  |
| Anhingidae |  |  |  |  |  |  |
|  | **Darter** | *Ahinga melanogaster* | C | NA | NA |  |
| Accipitridae |  |  |  |  |  |  |
|  | Black-winged Kite | *Elanus caeruleus* | C | NA | NA |  |
|  | Crested Serpent Eagle | *Spilornis cheela* | C | NA | NA |  |
| **Family** | **English name** | **Scientific Name** | **Diet^1^** | **Vert^2^** | **Mass** | **Source** |
|  | Crested Goshawk* | *Accipiter trivirgatus* | C | NA | NA |  |
|  | Shikra | *Accipiter badius* | C | mid | NA |  |
|  | Besra* | *Accipiter virgatus* | C | NA | NA |  |
|  | Black Eagle* | *Ictinaetus malayensis* | C | NA | NA |  |
|  | Changeable Hawk Eagle | *Nisaetus cirrhatus* | C | NA | NA |  |
|  | Legge’s Hawk Eagle | *Nisaetus kelaarti* | C | NA | NA |  |
| Falconidae |  |  |  |  |  |  |
|  | Common Kestrel | *Falco tinnunculus* | C | NA | NA |  |
| Rallidae |  |  |  |  |  |  |
|  | White-breasted Waterhen | *Amaurornis phoenicurus* | I | grass | 180 |  |
| Charadriidae |  |  |  |  |  |  |
|  | Red-wattled Lapwing | *Vanellus indicus* | C | NA | NA |  |
| Jacanidae |  |  |  |  |  |  |
|  | Bronze-winged Jacana | *Metopidius indicus* | I | grass | 155 |  |
| Scolopacidae |  |  |  |  |  |  |
|  | Common Sandpiper | *Actitis hypoleucos* | C | NA | NA |  |
| Laridae |  |  |  |  |  |  |
|  | **River Tern** | *Sterna aurantia* | C | NA | NA |  |
|  | Whiskered Tern | *Chlidonias hybrid* | C | NA | NA |  |
| Columbidae |  |  |  |  |  |  |
|  | Rock Pigeon | *Columba livia* | G | NA | NA |  |
|  | **Sri Lanka Wood Pigeon*** | *Columba torringtoniae* | F | NA | NA |  |
|  | Spotted Dove | *Spilopelia chinensis* | F | NA | NA |  |
|  | Emerald Dove | *Chalcophaps indica* | F | NA | NA |  |
|  | Grey-fronted Green Pigeon | *Treron affinis* | F | NA | NA |  |
|  | Sri Lanka Green Pigeon | *Treron pompadora* | F | NA | NA |  |
|  | Yellow-footed Green Pigeon | *Treron phoenicoptera* | F | NA | NA |  |
|  | Green Imperial Pigeon* | *Ducula aenea* | F | NA | NA |  |
|  | Mountain Imperial Pigeon* | *Ducula badia* | F | NA | NA |  |
| Psittacidae |  |  |  |  |  |  |
|  | Vernal Hanging Parrot* | *Loriculus vernalis* | F | NA | NA |  |
| **Family** | **English name** | **Scientific Name** | **Diet^1^** | **Vert^2^** | **Mass** | **Source** |
|  | Sri Lanka Hanging Parrot* | *Loriculus beryllinus* | F | NA | NA |  |
|  | Rose-ringed Parakeet | *Psittacula krameri* | F | NA | NA |  |
|  | Plum-headed Parakeet | *Psittacula cyanocephala* | F | NA | NA |  |
|  | Malabar Parakeet* | *Psittacula columboides* | F | NA | NA |  |
|  | Layard's Parrotkeet | *Psittacula calthorpae* | F | NA | NA |  |
| Cuculidae |  |  |  |  |  |  |
|  | **Green-billed Coucal*** | *Centropus chlororhychos* | I | under | 203 | EG, unpubl. data |
|  | Greater Coucal | *Centropus sinensis* | I | under | 283 |  |
|  | **Red-faced Malkoha*** | *Phaenicophaeus pyrrhocephalus* | I | canopy | NA |  |
|  | Blue-faced Malkoha* | *Phaenicophaeus viridirostris* | I | mid | 67 |  |
|  | Chestnut-winged Cuckoo* | *Clamator coromandus* | I | mid | 75 |  |
|  | Asian Koel | *Eudynamys scolopacea* | F | NA | NA |  |
|  | Bay-banded Cuckoo | *Cacomantis sonneratii* | I | mid | 34 |  |
|  | Drongo Cuckoo | *Surniculus lugubris* | I | canopy | NA |  |
|  | Common Hawk Cuckoo | *Hierococcyx varius* | I | mid | 103 |  |
|  | Indian Cuckoo | *Cuculus micropterus* | I | canopy | NA |  |
| Strigidae |  |  |  |  |  |  |
|  | Collared Scops Owl | *Otus lettia* | C | NA | NA |  |
|  | Brown Fishing Owl | *Ketupa zeylonensis* | C | NA | NA |  |
|  | Jungle Owlet* | *Glaucidium radiatum* | C | NA | NA |  |
|  | **Chestnut-backed Owlet*** | *Glaucidium castanonotum* | C | NA | NA |  |
| Podargidae |  |  |  |  |  |  |
|  | Sri Lanka Frogmouth* | *Batrachostomus moniliger* | I | mid | 54 |  |
| Hemiprocnidae |  |  |  |  |  |  |
|  | Crested Treeswift | *Hemiprocne coronata* | I | canopy | NA |  |
| Apodidae |  |  |  |  |  |  |
|  | Palm Swift | *Cypsiurus balasiensi* | I | aerial | NA |  |
| Trogonidae |  |  |  |  |  |  |
|  | Malabar Trogon* | *Harpactes fasciatus* | I | mid | 62 |  |
| Coraciidae |  |  |  |  |  |  |
|  | Dollarbird | *Eurystomus orientalis* | I | canopy | NA |  |
| **Family** | **English name** | **Scientific Name** | **Diet^1^** | **Vert^2^** | **Mass** | **Source** |
| Alcedinidae |  |  |  |  |  |  |
|  | Stork-billed Kingfisher | *Pelargopsis capensis* | C | NA | NA |  |
|  | White-throated Kingfisher | *Halcyon smyrnensis* | C | NA | NA |  |
|  | Common Kingfisher | *Alcedo atthis* | C | NA | NA |  |
|  | Pied Kingfisher | *Ceryle rudis* | C | NA | NA |  |
| Meropidae |  |  |  |  |  |  |
|  | Blue-tailed Bee-eater | *Merops philippinus* | I | canopy | NA |  |
|  | Chestnut-headed Bee-eater* | *Merops leschenaulti* | I | canopy | NA |  |
| Upupidae |  |  |  |  |  |  |
|  | Hoopoe | *Upupa epops* | I | grass | 72 |  |
| Bucerotidae |  |  |  |  |  |  |
|  | Malabar Grey Hornbill* | *Ocyceros griseus* | F | NA | NA |  |
|  | Sri Lanka Grey Hornbill | *Ocyceros gingalensis* | F | NA | NA |  |
| Megalaimidae |  |  |  |  |  |  |
|  | Brown-headed Barbet | *Megalaima zeylanica* | F | NA | NA |  |
|  | White-cheeked Barbet | *Megalaima viridis* | F | NA | NA |  |
|  | Yellow-fronted Barbet | *Megalaima flavifrons* | F | NA | NA |  |
|  | Malabar Barbet* | *Megalaima malabarica* | F | NA | NA |  |
|  | Ceylon Small Barbet | *Megalaima rubricapillus* | F | NA | NA |  |
|  | Coppersmith Barbet | *Megalaima haemacephala* | F | NA | NA |  |
| Picidae |  |  |  |  |  |  |
|  | Speckled Piculet* | *Picumnus innominatus* | I | mid | 10 |  |
|  | Heart-spotted Woodpecker | *Hemicircus canente* | I | mid | 44 |  |
|  | Brown-capped Woodpecker | *Dendrocopos nanus* | I | mid | 14 |  |
|  | White-bellied Woodpecker | *Dryocopus javensis* | I | mid | 272 |  |
|  | Lesser Yellownape* | *Picus chlorolophus* | I | mid | 66 |  |
|  | Streak-throated Woodpecker | *Picus xanthopygaeus* | I | mid | 100 |  |
|  | Common Flameback* | *Dinopium javanense* | I | mid | 73 |  |
|  | Black-rumped Flameback | *Dinopium benghalense* | I | mid | 100 |  |
|  | Greater Flameback* | *Chrysocolaptes lucidus* | I | mid | 142 |  |
|  | Crimson-backed Flameback | *Chrysocolaptes stricklandii* | I | mid | 234 |  |
| **Family** | **English name** | **Scientific Name** | **Diet^1^** | **Vert^2^** | **Mass** | **Source** |
|  | Rufous Woodpecker* | *Micropternus brachyurus* | I | mid | 108 |  |
| Pittadae |  |  |  |  |  |  |
|  | Indian Pitta* | *Pitta brachyura* | I | under | 56 |  |
| Tephrodornithidae |  |  |  |  |  |  |
|  | Bar-winged Flycatcher-Shrike | *Hemipus picatus* | I | canopy | NA |  |
|  | Malabar Woodshrike | *Tephrodornis sylvicola* | I | canopy | NA |  |
|  | Common Woodshrike | *Tephrodornis pondicerianus* | I | canopy | NA |  |
| Artamidae |  |  |  |  |  |  |
|  | Ashy Woodswallow | *Artamus fuscus* | I | canopy | NA |  |
| Aegithinidae |  |  |  |  |  |  |
|  | Common Iora | *Aegithina tiphia* | I | mid | 12 |  |
| Campephagidae |  |  |  |  |  |  |
|  | Large Cuckooshrike | *Coracina macei* | I | canopy | NA |  |
|  | Black-headed Cuckoo-Shrike | *Coracina melanoptera* | I | canopy | NA |  |
|  | Small Minivet | *Pericrocotus cinnamomeus* | I | canopy | NA |  |
|  | Orange Minivet* | *Pericrocotus flammeus* | I | canopy | NA |  |
| Laniidae |  |  |  |  |  |  |
|  | Brown Shrike | *Lanius cristatus* | I | grass | 34 |  |
|  | Long-tailed Shrike | *Lanius schach* | I | grass | 52 |  |
| Oriolidae |  |  |  |  |  |  |
|  | Indian Golden Oriole | *Oriolus kundoo* | I | canopy | NA |  |
|  | Black-naped Oriole | *Oriolus chinensis* | I | canopy | NA |  |
|  | Black-hooded Oriole | *Oriolus xanthornus* | I | canopy | NA |  |
| Dicuridae |  |  |  |  |  |  |
|  | Ashy Drongo | *Dicrurus leucophaeus* | I | canopy | NA |  |
|  | White-bellied Drongo | *Dicrurus caerulescens* | I | canopy | NA |  |
|  | Bronze Drongo | *Dicrurus aeneus* | I | canopy | NA |  |
|  | Sri Lanka Crested Drongo* | *Dicrurus lophorhinus* | I | mid | 78 | EG, unpubl. data |
|  | Greater Racket-tailed Drongo* | *Dicrurus paradiseus* | I | mid | 75 |  |
| Monarchidae |  |  |  |  |  |  |
|  | Black-naped Monarch | *Hypothymis azurea* | I | under | 11 |  |
| **Family** | **English name** | **Scientific Name** | **Diet^1^** | **Vert^2^** | **Mass** | **Source** |
|  | Asian Paradise Flycatcher | *Terpsiphone paradisi* | I | under | 18 |  |
| Corvidae |  |  |  |  |  |  |
|  | **Sri Lanka Magpie*** | *Urocissa ornata* | I | mid | 196 |  |
|  | Rufous Treepie | *Dendrocitta vagabunda* | C | NA | NA |  |
|  | White-bellied Treepie* | *Dendrocitta leucogastra* | C | NA | NA |  |
|  | House Crow | *Corvus splendens* | C | NA | NA |  |
|  | Large-billed Crow | *Corvus macrorhynchos* | C | NA | NA |  |
| Stenostiridae |  |  |  |  |  |  |
|  | Grey-headed Flycatcher | *Culicicapa ceylonensis* | I | mid | 8 |  |
| Paridae |  |  |  |  |  |  |
|  | Great Tit | *Parus major* | I | mid | 15 |  |
|  | Black-lored Tit | *Parus xanthogenys* | I | canopy | NA |  |
| Alaudidae |  |  |  |  |  |  |
|  | Rufous-winged Bushlark | *Mirafra affinis* | I | grass | 26 |  |
| Pycnonotidae |  |  |  |  |  |  |
|  | **Grey-headed Bulbul*** | *Pycnonotus priocephalus* | F | NA | NA |  |
|  | Black-capped Bulbul* | *Pycnonotus melanicterus* | F | NA | NA |  |
|  | Flame-throated Bulbul* | *Pycnonotus gularis* | F | NA | NA |  |
|  | Red-Whiskered Bulbul | *Pycnonotus jocosus* | F | NA | NA |  |
|  | Red-vented Bulbul | *Pycnonotus cafer* | F | NA | NA |  |
|  | **Yellow-eared Bulbul*** | *Pycnonotus penicillatus* | F | NA | NA |  |
|  | White-browed Bulbul | *Pycnonotus luteolus* | F | NA | NA |  |
|  | Yellow-browed Bulbul* | *Acritillas indica* | F | NA | NA |  |
|  | Black Bulbul | *Hypsipetes ganeesa* | F | NA | NA |  |
| Hirundinidae |  |  |  |  |  |  |
|  | Barn Swallow | *Hirundo rustica* | I | aerial | NA |  |
|  | Red-rumped Swallow | *Hirundo daurica* | I | aerial | NA |  |
|  | Sri Lanka Swallow | *Hirundo hyperythra* | I | aerial | NA |  |
| Phylloscopidae |  |  |  |  |  |  |
|  | Greenish Warbler | *Phylloscopus trochiloides* | I | mid | 8.3 |  |
|  | Large-billed Leaf Warbler* | *Phylloscopus magnirostris* | I | mid | 12 |  |
| **Family** | **English name** | **Scientific Name** | **Diet^1^** | **Vert^2^** | **Mass** | **Source** |
|  | Western Crowned Warbler* | *Phylloscopus occipitalis* | I | canopy | NA |  |
| Acrocephalidae |  |  |  |  |  |  |
|  | Blyth's Reed Warbler | *Acrocephalus dumetorum* | I | under | 11 |  |
|  | Thick-billed Warbler | *Iduna aedon* | I | under | 22 |  |
| Locustellidae |  |  |  |  |  |  |
|  | **Sri Lanka Bush Warbler*** | *Elaphrornis palliseri* | I | under | 9 |  |
| Cisticolidae |  |  |  |  |  |  |
|  | Grey-breasted Prinia | *Prinia hodgsonii* | I | grass | 6 |  |
|  | Ashy Prinia | *Prinia socialis* | I | grass | 8 |  |
|  | Plain Prinia | *Prinia inornata* | I | grass | 7 |  |
|  | Common Tailorbird | *Orthotomus sutorius* | I | under | 7.5 |  |
| Timaliidae |  |  |  |  |  |  |
|  | Indian Scimitar Babbler | *Pomatorhinus horsfieldii* | I | under | 43 |  |
|  | Sri Lanka Scimitar Babbler | *Pomatorhinus melanurus* | I | under | 43 |  |
|  | Dark-fronted Babbler | *Rhopocichla atriceps* | I | under | 16 |  |
| Pellorneidae |  |  |  |  |  |  |
|  | Brown-cheeked Fulvetta | *Alcippe poioicephala* | I | under | 21 |  |
|  | Puff-throated Babbler* | *Pellorneum ruficeps* | I | under | 26 |  |
|  | Brown-capped Babbler | *Pellorneum fuscocapillum* | I | under | 30 |  |
| Leiothrichidae |  |  |  |  |  |  |
|  | Common Babbler* | *Turdoides caudate* | I | grass | 34 |  |
|  | Rufous Babbler | *Turdoides subrufa* | I | under | 68 |  |
|  | Jungle Babbler | *Turdoides striatus* | I | under | 66 |  |
|  | **Orange-billed Babbler*** | *Turdoides rufescens* | I | mid | 66 | EG, unpubl. data |
|  | Yellow-billed Babbler | *Turdoides affinis* | I | grass | 66 | *Turdoides striata* |
|  | **Ashy-headed Laughingthrush*** | *Garrulax cinereifrons* | I | under | 70 |  |
|  | Wynad's Laughing-thrush* | *Garrulax delesserti* | I | under | 92 | *Garrulax gularis* |
| Sylviidae |  |  |  |  |  |  |
|  | Yellow-eyed Babbler | *Chrysomma sinense* | I | under | 16 |  |
| Zosteropidae |  |  |  |  |  |  |
|  | Oriental White-eye | *Zosterops palpebrosus* | I | canopy | NA |  |
| **Family** | **English name** | **Scientific Name** | **Diet^1^** | **Vert^2^** | **Mass** | **Source** |
|  | Sri Lanka White-eye | *Zosterops ceylonensis* | I | canopy | NA |  |
| Irenidae |  |  |  |  |  |  |
|  | Asian Fairy Bluebird* | *Irena puella* | F | NA | NA |  |
| Sittidae |  |  |  |  |  |  |
|  | Velvet-fronted Nuthatch | *Sitta frontalis* | I | mid | 16 |  |
| Sturnidae |  |  |  |  |  |  |
|  | **Sri Lanka Myna** | *Gracula ptilogenys* | F | NA | NA |  |
|  | Southern Hill Myna | *Gracula indica* | F | NA | NA |  |
|  | Jungle Myna | *Acridotheres fuscus* | I | mid | 83 |  |
|  | Common Myna | *Acridotheres tristis* | I | grass | 116 |  |
|  | **White-faced Starling*** | *Sturnus albofrontatus* | F | NA | NA |  |
|  | Chestnut-tailed Starling | *Sturnus malabaricus* | I | canopy | NA |  |
| Turdidae |  |  |  |  |  |  |
|  | **Sri Lanka Whistling Thrush*** | *Myophonus blighi* | I | under | 117 | *Myophonus horsfieldii* |
|  | Malabar Whistling Thrush | *Myophonus horsfieldii* | I | under | 117 |  |
|  | Pied Thrush | *Geokichla wardii* | I | under | 59 |  |
|  | Orange-headed Thrush | *Geokichla citrina* | I | under | 53 |  |
|  | **Spot-winged Thrush*** | *Geokichla spiloptera* | I | under | 70 |  |
|  | **Sri Lanka Scaly Thrush*** | *Zoothera imbricata* | I | under | 104 |  |
|  | Indian Blackbird | *Turdus simillimus* | I | under | 75 |  |
| Muscicapidae |  |  |  |  |  |  |
|  | Indian Blue Robin | *Luscinia cyane* | I | under | 17 |  |
|  | Oriental Magpie Robin | *Copsychus saularis* | I | grass | 36 |  |
|  | Indian Robin | *Saxicoloides fulicatus* | I | grass | 17 |  |
|  | Pied Bushchat | *Saxicola caprata* | I | grass | 15 |  |
|  | Blue-capped Rock Thrush | *Monticola cinclorhynchus* | I | under | 36 |  |
|  | Asian Brown Flycatcher | *Muscicapa dauurica* | I | mid | 10 |  |
|  | Brown-breasted Flycatcher* | *Muscicapa muttui* | I | mid | 12 |  |
|  | Rusty-tailed Flycatcher | *Muscicapa ruficauda* | I | under | 13 |  |
|  | **Kashmir Flycatcher** | *Ficedula subrubra* | I | under | 10 |  |
|  | **Dull-blue Flycatcher** | *Eumyias sordida* | I | under | 16 | *Eumyias albicaudatus* |
| **Family** | **English name** | **Scientific Name** | **Diet^1^** | **Vert^2^** | **Mass** | **Source** |
|  | Verditer Flycatcher | *Eumyias thalassinus* | I | canopy | NA |  |
|  | **Nilgiri Flycatcher** | *Eumyias albicaudata* | I | under | 16 |  |
|  | White-bellied Blue Flycatcher* | *Cyornis pallipes* | I | under | 19 |  |
|  | Tickell's Blue Flycatcher | *Cyornis tickelliae* | I | under | 15 |  |
| Chloropseidae |  |  |  |  |  |  |
|  | Blue-winged Leafbird | *Chloropsis cochinchinensis* | F | NA | NA |  |
|  | Gold-fronted Leafbird* | *Chloropsis aurifrons* | F | NA | NA |  |
| Dicaeidae |  |  |  |  |  |  |
|  | **White-throated Flowerpecker** | *Dicaeum vincens* | F | NA | NA |  |
|  | Pale-billed Flowerpecker | *Dicaeum erythrorhynchos* | F | NA | NA |  |
|  | Plain Flowerpecker | *Dicaeum minullum* | F | NA | NA |  |
| Nectariniidae |  |  |  |  |  |  |
|  | Purple-rumped Sunbird | *Leptocoma zeylonica* | N | NA | NA |  |
|  | Crimson-backed Sunbird | *Leptocoma minima* | N | NA | NA |  |
|  | Purple Sunbird | *Cinnyris asiaticus* | N | NA | NA |  |
|  | Long-billed Sunbird | *Cinnyris lotenius* | N | NA | NA |  |
|  | Little Spiderhunter* | *Arachnothera longirostra* | N | NA | NA |  |
| Passeridae |  |  |  |  |  |  |
|  | House Sparrow | *Passer domesticus* | G | NA | NA |  |
| Estrildidae |  |  |  |  |  |  |
|  | White-rumped Munia | *Lonchura striata* | G | NA | NA |  |
|  | Scaly-breasted Munia | *Lonchura punctulata* | G | NA | NA |  |
|  | Black-throated Munia | *Lonchura kelaarti* | G | NA | NA |  |
|  | Black-headed Munia | *Lonchura malacca* | G | NA | NA |  |
| Motacillidae |  |  |  |  |  |  |
|  | Forest Wagtail | *Dendronanthus indicus* | I | under | 16 |  |
|  | Grey Wagtail | *Motacilla cinerea* | I | under | 18 |  |
|  | Paddy Pipit | *Anthus rufulus* | I | grass | 20 |  |

^1^ = Abbreviations for diets: I = invertebrate carnivore, C = vertebrate carnivore, F = frugivore; N = nectarivore, G = granivore.

^2^ = Species considered in analysis “understorey” if classified as either terrestrial or understorey; similarly, considered “canopy” if classified as either midstory or canopy.
